# Wheat Class I TCP Transcription Factor TaTCP15 Positively Regulates Cutin and Cuticular Wax Biosynthesis

**DOI:** 10.3390/biom16020192

**Published:** 2026-01-27

**Authors:** Linzhu Fang, Xiaoyu Wang, Haoyu Li, Jiao Liu, Pengfei Zhi, Cheng Chang

**Affiliations:** College of Life Sciences, Qingdao University, Qingdao 266071, China

**Keywords:** wheat, cutin, cuticular wax, TCP transcription factor, transcriptional regulation

## Abstract

Cutin matrices and wax mixtures are major components of lipophilic cuticles, shielding plant tissues from stressful environments. Identifying the key regulators governing biosynthesis of cutin and cuticular wax in bread wheat (*Triticum aestivum* L.) could contribute to wheat breeding for stress resistance. In this study, we reported that the wheat class I TCP transcription factor TaTCP15 positively regulates cutin and cuticular wax biosynthesis. The CYP86A family cytochrome P450 enzymes, TaCYP86A2 and TaCYP86A4, were characterized as essential components of wheat cutin biosynthetic machinery. Wheat transcription factor TaSHN1 targets *TaCYP86A2*, *TaCYP86A4*, and wax biosynthesis gene *TaECR* and recruits the mediator subunit TaCDK8 to activate these genes’ transcription. Furthermore, we demonstrated that *TaSHN1* gene transcription is directly activated by the transcription factor TaTCP15. Expression of *TaSHN1*, *TaCYP86A2*, *TaCYP86A4*, and *TaECR* genes, as well as cutin and wax accumulation, was attenuated by silencing of the *TaTCP15* gene. Collectively, these findings suggest that wheat class I TCP transcription factor TaTCP15 positively regulates cutin and cuticular wax biosynthesis, probably via directly targeting the *TaSHN1* gene and upregulating *TaCYP86A2*, *TaCYP86A4*, and *TaECR* expression, providing valuable information for developing wheat plants with improved cuticle-associated traits.

## 1. Introduction

During the transition from aquatic to terrestrial habitats, early land plants acquired a hydrophobic skin, the cuticle, to shield plant tissues from desiccation [1]. The lipophilic cuticle not only covers the aboveground organs, like leaves, fruits, flowers, and nonwoody stems, but also temporally coats the underground root tips [2]. As an evolutionary innovation, the waxy cuticle mainly functions as a barrier to restrict plant non-stomatal water loss, screen ultraviolet (UV) radiation, attenuate mechanical damage, impair gas exchanges and heat transference between plant tissues and surrounding environments, and protect plant tissues from pathogen infections and pest infestations [3,4,5,6]. A plethora of developmental processes, such as organ separation, seedling establishment, and lateral root formation, are also affected by the cuticle [7]. Cutin matrices and wax mixtures are two major lipophilic components of the cuticle. As an organic solvent-insoluble fraction, cutin consists of crosslinked polyesters of oxygenated C16 and C18 fatty acids, like ω-hydroxy fatty acids (FAs), polyhydroxy FAs, and dicarboxylic acids [8]. In contrast, cuticular waxes are organic solvent-extractable and predominantly composed of very long-chain (VLC, ≥C20) fatty acids and derivatives, including VLC alcohols, alkanes, aldehydes, ketones, and alkyl esters [9,10].

As described by prior reviews, biosynthesis of plant cutin and wax is extensively studied in the dicot model plant *Arabidopsis thaliana* [11]. For cutin biosynthesis, C16 and C18 fatty acyl-CoAs generated by long-chain acyl-coenzyme A synthases (LACS) undergo oxidation and acyl transfer reactions catalyzed by CYP86A and CYP77A family cytochrome P450 enzymes, the HOTHEAD protein, and glycerol-3-phosphate (G3P) acyltransferase (GPAT) enzymes to form the cutin precursors monoacylglycerols (MAGs) [12,13,14,15,16,17,18,19]. For cuticular wax biosynthesis, these C16 and C18 fatty acyl-CoAs are modified by fatty acid elongase (FAE) enzymes to generate elongated VLC acyl-CoAs [20,21,22,23,24,25,26,27,28]. Cuticular wax components are concerted from VLC acyl-CoAs via the alkane-forming pathway and alcohol-forming pathway [29,30,31,32,33,34,35,36,37]. These synthesized wax components and cutin precursors are then exported to the extracellular cuticular region [38,39,40,41,42,43,44,45,46]. However, the mechanism underlying cutin and wax biosynthesis in agronomically important crops like bread wheat (*Triticum aestivum* L.) remains poorly understood.

In this study, we reported that the wheat class I TCP transcription factor TaTCP15 directly activates transcription of *TaSHN1*, a transcriptional activator gene of wheat cutin and wax biosynthesis, thereby positively regulating wheat cuticle biosynthesis. Cutin biosynthesis genes *TaCYP86A2* and *TaCYP86A4* and wax biosynthesis gene *TaECR* were identified as target genes of *TaSHN1*. This study revealed a novel regulatory mechanism underlying wheat cuticle biosynthesis and provided valuable information for developing wheat plants with improved cuticle-associated traits.

## 2. Materials and Methods

### 2.1. Plant Materials and Growth Conditions

After surface disinfection, as described in [47], seeds of winter wheat cultivar Yannong 999 were germinated in glass dishes containing sterile Hoagland solution, and seedlings were cultivated in climate chambers under a 16 h light/8 h dark, 20 °C/18 °C day/night cycle.

### 2.2. Accession Numbers

Sequence data of the genes identified in this research can be found under accession numbers *TaCYP86A4-2A* (*TraesCS2A02G407500*), *TaCYP86A4-2B* (*TraesCS2B02G425400*), *TaCYP86A4-2D* (*TraesCS2D02G404600*), *TaTCP15-6A* (*TraesCS6A02G306500*), *TaTCP15-6B* (*TraesCS6B02G334900*), and *TaTCP15-6D* (*TraesCS6D02G285600*).

### 2.3. Gene Silencing Assay

A barley stripe mosaic virus-induced gene silencing (BSMV-VIGS) assay was conducted to silence *TaCYP86A2*, *TaCYP86A4*, *TaECR*, *TaSHN1*, *TaCDK8*, or *TaTCP15* genes encoding TaCYP86A2, TaCYP86A4, TaECR, TaSHN1, TaCDK8, or TaTCP15 proteins. Briefly, antisense (as) fragments from the coding regions of *TaCYP86A4* and *TaTCP15* genes were amplified using the primers listed in Supplementary Table S1 and cloned into the pCa-γbLIC vector to generate BSMV-*TaCYP86A4as* and BSMV-*TaTCP15as* constructs. Constructs BSMV-*TaCYP86A2as*, BSMV-*TaECRas*, BSMV-*TaSHN1as*, and BSMV-*TaCDK8as* were derived from previous studies [48,49,50]. BSMV-VIGS assays were performed as previously described [51].

### 2.4. Gene Expression Analysis

RT-qPCR assays were performed to analyze the expression levels of *TaCYP86A2*, *TaECR*, *TaSHN1*, *TaCDK8*, or *TaTCP15* gene using the ABI step-one real-time PCR system. Accumulation of *TaCYP86A2*, *TaECR*, *TaSHN1*, *TaCDK8*, or *TaTCP15* transcripts was analyzed using the primers listed in Supplementary Table S1, and the geometric mean of two housekeeping genes, *TaACTIN* and *TaGADPH*, was employed for RT-qPCR analysis.

### 2.5. Transcriptional Activation Analysis

For transcriptional activation analysis, *TaSHN1*, *TaCDK8*, and *TaTCP15* coding regions were amplified using the primers listed in Supplementary Table S1 and cloned into the vector pIPKb004. Wild-type and mutant versions of *TaCYP86A2*, *TaECR*, and *TaSHN1* promoters were ligated into the vector 5XGAL4-LUC; 5XGAL4-LUC (Addgene, #24343) was described by Potter et al. [52]. For wheat protoplast preparation, 7-day-old wheat leaves were harvested and digested in a Cellulase and Macerozyme solution for 6 h. After washing three times with W5 solution containing 150 mM NaCl, 120 mM CaCl_2_, 5 mM KCl, and 2 mM MES, wheat protoplasts were kept in MMG solution containing 0.4 M mannitol, 15 mM MgCl_2_, and 4 mM MES, pH 5.7, on ice for transformation with reporter and effector constructs. Wheat protoplast transfection was conducted as described in [50].

### 2.6. Cuticular Lipid Composition Analysis

The cuticular lipid composition analysis was performed as described in [14,53]. Wheat leaves were delipidated by chloroform (Merck, Rahway, NJ, USA), and the wax lipid extracts were derivatized and analyzed by capillary gas chromatography (GC) and a flame ionization detector (FID) with a mass spectrometer. For the cutin composition analysis, delipidated wheat leaf samples were depolymerized, and the generated methyl esters were extracted with dichloromethane and subjected to GC analysis.

### 2.7. Wheat Leaf Cuticle Permeability Analysis

Rates of water loss and chlorophyll leaching were measured to analyze the cuticle permeability of wheat leaves in plants with the silenced genes, as described in [54,55]. For the water loss rate analysis, the weights of detached leaves were measured per hour for 12 h. For the chlorophyll leaching assay, the detached leaves were soaked in the 80% ethanol, and the chlorophyll concentration in the liquid was measured with a spectrophotometer every hour for 12 h.

### 2.8. Analyses of Protein Enrichment at Gene Promoter Regions

ChIP assays were performed to analyze the enrichment of TaSHN1, TaCDK8, and TaTCP15 proteins at promoter regions of their target genes *TaCYP86A2*, *TaECR*, and *TaSHN1*. The ChIP assays were carried out as described in [49,50]. DNA recovery from the ChIP assays was quantified as a percentage of input. qPCR was performed using the primers listed in Supplementary Table S1, as described above.

### 2.9. Statistical Analysis

Three technical replicates were analyzed per assay, and data are presented as mean ± standard deviation. Each assay was repeated with three biological replicates using separately prepared samples. Student’s *t*-test was performed to statistically compare two groups, whereas One-way ANOVA with Duncan’s post hoc test was conducted to analyze three or more groups.

## 3. Results

### 3.1. Characterization of TaCYP86A2 and TaCYP86A4 Genes Essential for Wheat Cutin Biosynthesis

**Figure 1:**
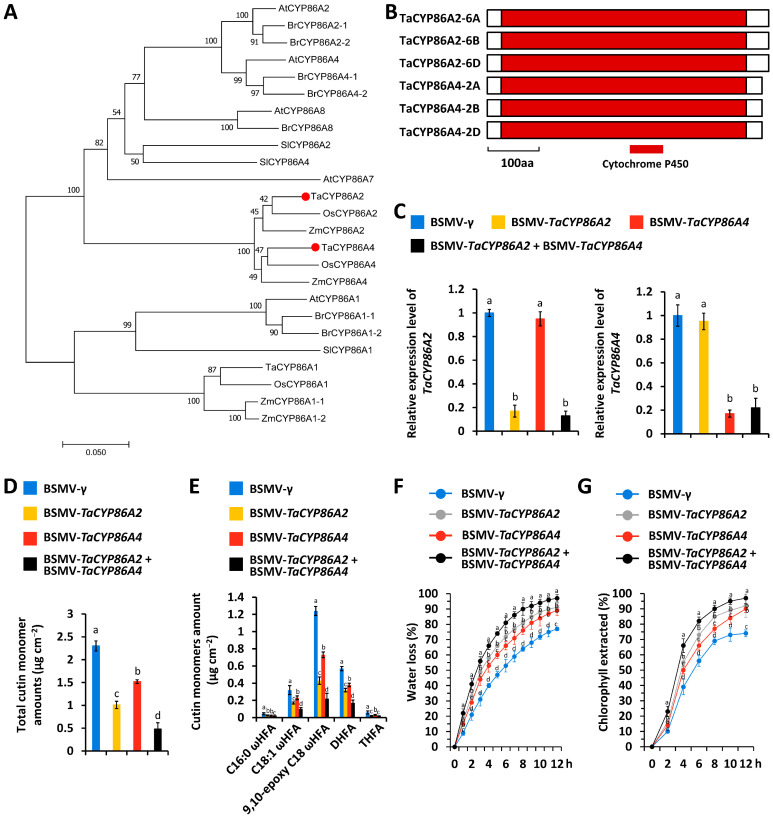
Functional characterization of wheat *TaCYP86A2* and *TaCYP86A4* genes in cutin biosynthesis. (**A**) Phylogenetic tree reconstruction of the CYP86A2 and CYP86A4 proteins identified from Arabidopsis (At), tomato (Sl), *Brassica rapa* (Br), rice (Os), maize (Zm), and bread wheat (Ta). TaCYP86A2-6D (TraesCS6D02G227200) and TaCYP86A4-2D (TraesCS2D02G404600) were employed as representatives of TaCYP86A2 and TaCYP86A4 and indicated with red circles. (**B**) Domain structure of wheat TaCYP86A2 and TaCYP86A4 proteins. (**C**) qRT-PCR analysis of *TaCYP86A2* and *TaCYP86A4* expression levels in wheat leaves with silenced *TaCYP86A2* (BSMV-*TaCYP86A2as*), *TaCYP86A4* (BSMV-*TaCYP86A4as*), or *TaCYP86A2*, and *TaCYP86A4* (BSMV-*TaCYP86A2as* + BSMV-*TaCYP86A4as*). (**D**) Total cutin and cuticular wax amounts in wheat leaves with silenced genes *TaCYP86A2*, *TaCYP86A4*, or *TaCYP86A2* and *TaCYP86A4*. (**E**) Cutin monomer accumulation in wheat leaves with silenced genes *TaCYP86A2*, *TaCYP86A4*, or *TaCYP86A2* and *TaCYP86A4*. (**F**) Water loss rates and (**G**) chlorophyll extraction levels analyzed in wheat leaves with silenced *TaCYP86A2*, *TaCYP86A4*, or *TaCYP86A2* and *TaCYP86A4*. BSMV-γ (BSMV empty vector) was employed as the negative control, and data were statistically analyzed by One-way ANOVA analysis (different letters represent *p* < 0.05).

Arabidopsis AtCYP86A2 and AtCYP86A4 are essential components of cutin biosynthetic machinery [19]. We first employed Arabidopsis AtCYP86A2 (*At4g00360*) and AtCYP86A4 (*At1g01600*), two essential components of cutin biosynthetic machinery, as queries to search the reference genome of allohexaploid bread wheat (data source: International Wheat Genome Sequencing Consortium, https://wheat-urgi.versailles.inra.fr/Seq-Repository/Assemblies (accessed on 1 July 2024) and identified *TaCYP86A2* and *TaCYP86A4* as the homologs of Arabidopsis *AtCYP86A2* and *AtCYP86A4* (Figure 1A). Three *TaCYP86A2* genes, separately located on wheat chromosomes 6A, 6B, and 6D, were designated as *TaCYP86A2-6A* (*TraesCS6A02G244900*), *TaCYP86A2-6B* (*TraesCS6B02G279400*), and *TaCYP86A2-6D* (*TraesCS6D02G227200*). *TaCYP86A4-2A* (*TraesCS2A02G407500*), *TaCYP86A4-2B* (*TraesCS2B02G425400*), and *TaCYP86A4-2D* (*TraesCS2D02G404600*) were separately identified from wheat chromosomes 2A, 2B, and 2D. As shown in Figure 1B, the cytochrome P450 domain was identified from the central part of these six TaCYP86A2 and TaCYP86A4 proteins.

To examine the regulation of wheat cutin biosynthesis by *TaCYP86A2* and *TaCYP86A4* genes, we separately silenced all endogenous *TaCYP86A2* or *TaCYP86A4* genes by BSMV-VIGS (Figure 1C). As shown in Figure 1D, single silencing of *TaCYP86A2* or *TaCYP86A4*, or co-silencing *TaCYP86A2* and *TaCYP86A4*, resulted in a significant reduction in the cutin accumulation on the wheat leaves. Major cutin monomers, including 16-hydroxy-hexadecanoic acid (C16:0 ωHFA), 18-hydroxy-octadec-9-enoic acid (C18:1 ωHFA), 9,10-epoxy 18-hydroxy-octadecanoic acid (9,10-epoxy C18 ωHFA), 9(10), 16-dihydroxy-hexadecanoic acid (DHFA), and 9,10,18-trihydroxy-octadecanoic acid (THFA), all showed a significant reduction in the wheat leaves with silenced genes *TaCYP86A2* or/and *TaCYP86A4* (Figure 1E). Measurement of the water loss rate and chlorophyll leaching confirmed that cuticle permeability was enhanced on the wheat leaves with silenced genes *TaCYP86A2* or/and *TaCYP86A4* (Figure 1F,G). These results suggest that wheat TaCYP86A2 and TaCYP86A4 proteins play key roles in wheat cutin biosynthesis.

### 3.2. Transcriptional Regulation of Cutin Biosynthesis Genes TaCYP86A2 and TaCYP86A4 and Wax Biosynthesis Gene TaECR by Transcription Factor TaSHN1 and Mediator Subunit TaCDK8

**Figure 2:**
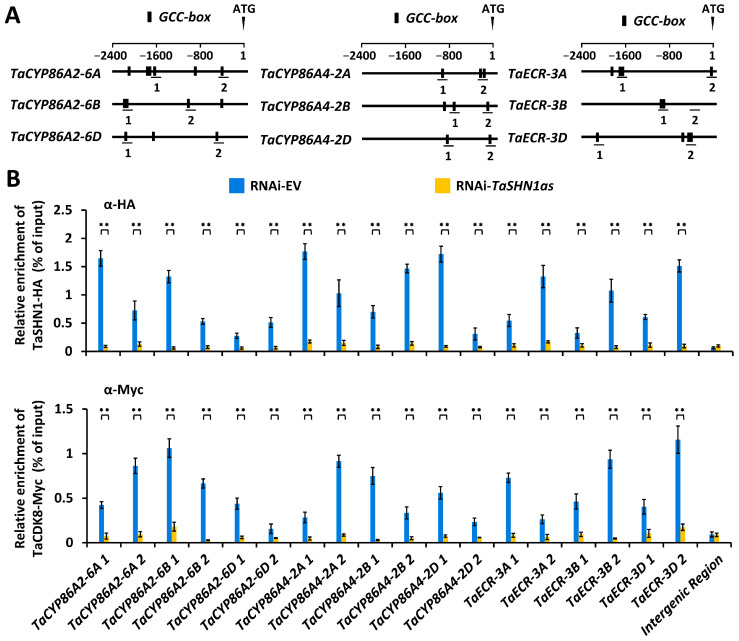
Analysis of TaSHN1 and TaCDK8 enrichment at promoter regions of TaCYP86A2, TaCYP86A4, and TaECR. (**A**) Schematic diagram of TaCYP86A2, TaCYP86A4, and TaECR promoter structures. GCC box cis-elements are shown as black boxes. Promoter regions subjected to ChIP-qPCR analysis are labeled with numbers. (**B**) ChIP-qPCR analysis of TaSHN1 and TaCDK8 occupancy at TaCYP86A2, TaCYP86A4, and TaECR promotes. For (**B**), data were statistically analyzed by Student’s *t*-test (** represents *p* < 0.01).

Multiple *GCC*-box cis-elements were identified in the promoter regions of cutin biosynthesis genes *TaCYP86A2* and *TaCYP86A4* and wax biosynthesis gene *TaECR* (Figure 2A). As reported by Kong and Chang [49], TaSHN1-6A, TaSHN1-6B, and TaSHN1-6D proteins encoded by allelic *TaSHN1* genes shared over 99% amino acid sequence identity; therefore, we chose TaSHN1-6A as a representative TaSHN1 in the following experiments. To analyze the potential enrichment of TaSHN1 and TaCDK8 proteins at *TaCYP86A2*, *TaCYP86A4*, and *TaECR* promoters, we expressed TaSHN1-HA and TaCDK8-Myc proteins in wheat protoplasts and analyzed the distribution of TaSHN1-HA and TaCDK8-Myc at *TaCYP86A2*, *TaCYP86A4*, and *TaECR* promoters by the chromatin immunoprecipitation (ChIP) assay (Figure 2A). As shown in Figure 2B, fragments of *TaCYP86A2*, *TaCYP86A4*, and *TaECR* promoters were observed to be co-immunoprecipitated with the TaSHN1-HA or TaCDK8-Myc protein, indicating that TaSHN1 and TaCDK8 associated with *TaCYP86A2*, *TaCYP86A4*, and *TaECR* promoters in bread wheat. Notably, enrichment levels of TaCDK8-Myc at *TaCYP86A2*, *TaCYP86A4*, and *TaECR* promoters were significantly reduced by knock-down of *TaSHN1* genes, suggesting that transcription factor TaSHN1 could recruit mediator subunit TaCDK8 to the promoter regions of cutin biosynthesis genes *TaCYP86A2* and *TaCYP86A4* and wax biosynthesis gene *TaECR* (Figure 2B).

**Figure 3:**
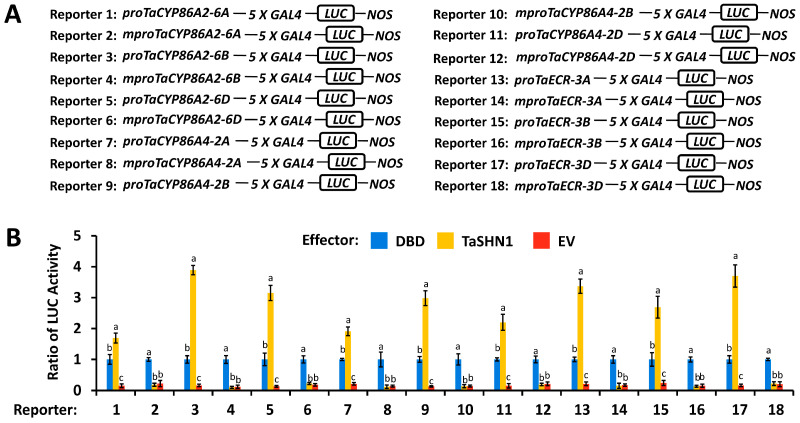
Analysis of transcriptional activation of *TaCYP86A2*, *TaCYP86A4*, and *TaECR* promoters by TaSHN1. (**A**) Schematic diagram of the reporter constructs containing wild-type (pro) or mutated (mpro) promoter fragments of *TaCYP86A2*, *TaCYP86A4*, and *TaECR* genes. (**B**) Transactivation of *TaCYP86A2*, *TaCYP86A4*, and *TaECR* genes by TaSHN1 in wheat protoplasts. Data were statistically analyzed by One-way ANOVA (different letters represent *p* < 0.05).

**Figure 4:**
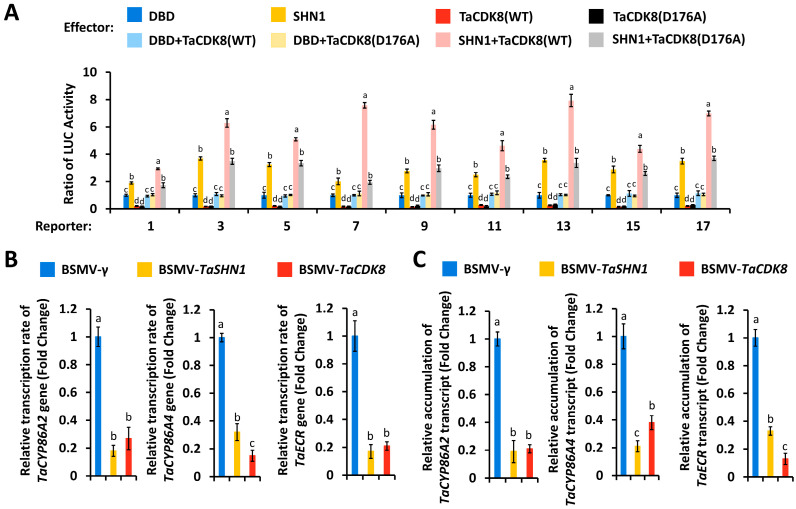
Analysis of transcriptional activation of *TaCYP86A2*, *TaCYP86A4*, and *TaECR* promoters by the TaCDK8-TaSHN1 module. (**A**) Effect of TaCDK8 on the TaSHN1-mediated transactivation of *TaCYP86A2*, *TaCYP86A4*, and *TaECR* promoters in wheat protoplasts. A schematic diagram of the reporter constructs is shown in Figure 3A. Nuclear run-on (**B**) and RT-qPCR (**C**) analysis of *TaCYP86A2*, *TaCYP86A4*, and *TaECR* transcription in the wheat leaves with silenced genes *TaSHN1* and *TaCDK8*. BSMV-γ was employed as the negative control. For (**A**–**C**), data were statistically analyzed by One-way ANOVA (different letters represent *p* < 0.05).

As demonstrated by Kong and Chang [49], TaSHN1 functions as a transcriptional activator. We next asked whether transcriptional activator TaSHN1 could directly trans-activate *TaCYP86A2*, *TaCYP86A4*, and *TaECR* promoters. We performed the wheat leaf protoplast transfection assay, in which the *LUC* reporter gene, driven by *TaCYP86A2*, *TaCYP86A4*, and *TaECR* promoters, was transfected with effector constructs over-expressing TaSHN1 proteins and a control plasmid. Wild-type promoter regions of *TaCYP86A2*, *TaCYP86A4*, and *TaECR* genes harboring the cis-elements *GCC*-box, and *TaCYP86A2*, *TaCYP86A4*, and *TaECR* promoter mutants containing mutated *GCC*-box cis-elements, were employed. As shown in Figure 3A,B, expression of *TaSHN1* resulted in a significant increase in the LUC activity obtained from LUC reporters containing wild-type *TaCYP86A2*, *TaCYP86A4*, and *TaECR* promoters. In contrast, the LUC activity obtained from *TaCYP86A2*, *TaCYP86A4*, and *TaECR* promoter mutants containing mutated *GCC*-box cis-elements was not significantly affected by over-accumulated TaSHN1 proteins, suggesting that transcriptional activator TaSHN1 directly binds to the promoter regions of *TaCYP86A2*, *TaCYP86A4*, and *TaECR* genes by recognizing the *GCC*-box cis-elements and stimulates their transcription (Figure 3A,B). Compared with the LUC activity obtained from the expression of TaSHN1, coexpression of TaSHN1 with wild-type TaCDK8 led to a further increase in the LUC activity obtained from the reporter gene driven by *TaCYP86A2*, *TaCYP86A4*, and *TaECR* promoters to above 3.29, but coexpression of TaSHN1 with the TaCDK8(D176A) mutant with defective kinase activity failed to affect the LUC activity (Figure 4A). Consistent with this, silencing of *TaSHN1* or *TaCDK8* resulted in a significant reduction in the transcription rates and expression levels of *TaCYP86A2*, *TaCYP86A4*, and *TaECR* genes (Figure 4B,C). Together, these results indicate that transcription factor TaSHN1 could recruit mediator kinase subunit TaCDK8 to activate transcription of cutin biosynthesis genes *TaCYP86A2* and *TaCYP86A4* and wax biosynthesis gene *TaECR*.

### 3.3. Identification of Transcription Factor TaTCP15 as a Transcriptional Regulator of the TaSHN1 Gene

**Figure 5:**
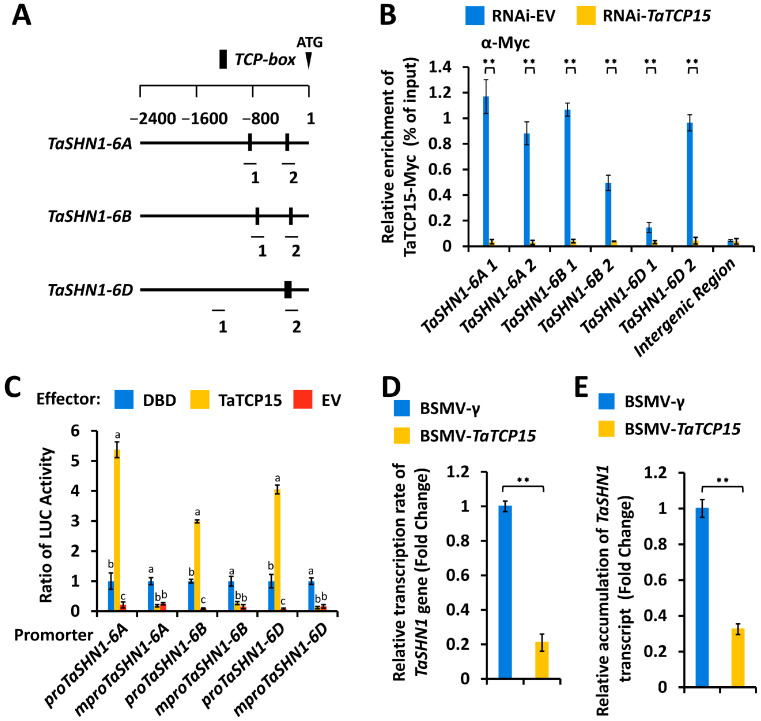
Transcriptional regulation of *TaSHN1* genes by transcription factor TaTCP15. (**A**) Schematic diagram of *TaSHN1* promoter structures. (**B**) ChIP-qPCR analysis of TaTCP15 occupancy at *TaSHN1* promoters. A fragment of the intergenic region was employed as the negative control. (**C**) Transactivation of the *TaSHN1* gene by TaTCP15 in wheat protoplast cells transfected with the indicated pairs of effectors and *LUC* reporter. Nuclear run-on (**D**) and RT-qPCR (**E**) analysis of *TaSHN1* transcription in the wheat leaves with silenced gene *TaTCP15*. BSMV-γ was employed as the negative control. For (**B**,**D**,**E**), three technical replicates per treatment were statistically analyzed by Student’s *t*-test (** represents *p* < 0.01). For (**C**), three technical replicates per treatment were statistically analyzed by One-way ANOVA analysis (different letters represent *p* < 0.05).

Multiple *TCP box* cis-elements were identified from TaSHN1 promoter regions (Figure 5A). Arabidopsis class I TCP transcription factor AtTCP15 could recognize the *TCP box* cis-element and directly activate *AtSHN1* transcription [50]. To analyze the potential enrichment of wheat TCP15 proteins at *TaSHN1* promoters, we employed Arabidopsis AtTCP15 (*At1g69690*) as a query to search the wheat genome (data source: International Wheat Genome Sequencing Consortium, https://wheat-urgi.versailles.inra.fr/Seq-Repository/Assemblies) and identified *TaTCP15-6A* (*TraesCS6A02G306500*), *TaTCP15-6B* (*TraesCS6B02G334900*), and *TaTCP15-6D* (*TraesCS6D02G285600*) from wheat chromosomes 6A, 6B, and 6D. Considering that the predicted TaTCP15-6A, TaTCP15-6B, and TaTCP15-6D proteins shared above 95% amino acid sequence identity, TaTCP15-6A was selected as a representative TaTCP15 in the following experiments. We expressed TaTCP15-Myc in wheat protoplasts and performed a ChIP assay to characterize the potential occupancy of the TaTCP15-Myc protein at *TaSHN1* promoters (Figure 5A). As shown in Figure 5B, *TaSHN1* promoter fragments were found to be immunoprecipitated with the antibody against TaTCP15-Myc, indicating that transcription factor TaTCP15 enriches at the *TCP box* cis-element regions in *TaSHN1* promoters in wheat cells.

As shown in Supplementary Figure S1, over-accumulation of DBD-TaTCP15 proteins resulted in a significant increase in LUC activity, suggesting that TaTCP15 functions as a transcriptional activator. We next asked whether transcriptional activator TaTCP15 could directly bind to *TaSHN1* promoters and activate their transcription. In the wheat leaf protoplast transfection assay, LUC reporters containing promoter regions of *TaSHN1-6A*, *TaSHN1-6B*, or *TaSHN1-6D* genes were transfected with effectors construct over-expressing TaTCP15 proteins and a control plasmid. Wild-type promoter regions of *TaSHN1-6A*, *TaSHN1-6B*, or *TaSHN1-6D* genes harboring the *TCP box* cis-element, and *TaSHN1-6A*, *TaSHN1-6B*, or *TaSHN1-6D* promoter mutants containing mutated *TCP box* cis-elements, were employed. As shown in Figure 5C, over-accumulation of TaTCP15 proteins resulted in a significant increase in LUC activity in the LUC reporters containing wild-type *TaSHN1* promoters but not in the LUC reporters harboring mutated *TaSHN1* promoters, suggesting that TaTCP15 could directly bind to *TaSHN1* promoters by recognizing the *TCP box* cis-element and activate their transcriptions (Figure 5C). As shown in Figure 5D, silencing the TaTCP15 gene resulted in a significant decrease in the transcription rates of the TaSHN1 gene. Consistent with this, accumulation levels of the TaSHN1 gene transcript decreased significantly in wheat leaves with silenced gene *TaTCP15*, compared with BSMV-γ (Figure 5E). These results collectively suggested that transcriptional activator TaTCP15 could be enriched at *TaSHN1* promoters by recognizing the *TCP box* cis-element and directly activating *TaSHN1* transcription in wheat cells.

### 3.4. Functional Characterization of the TaTCP15 Gene in Cutin and Wax Biosynthesis

**Figure 6:**
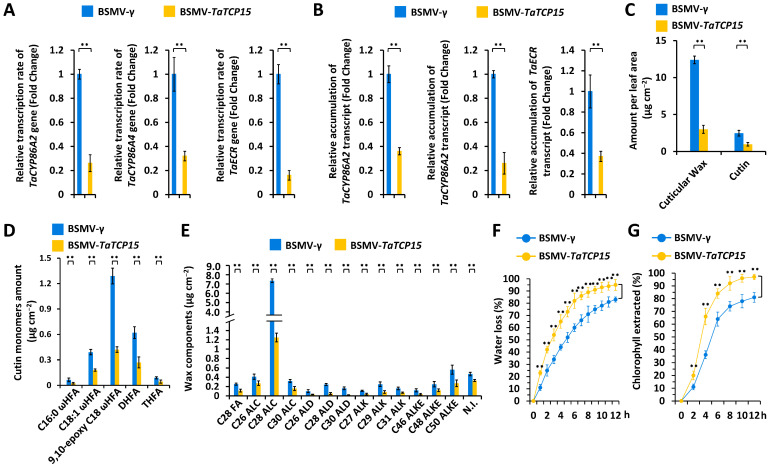
Functional analyses of wheat *TaTCP15* genes in cuticular wax and cutin accumulation. Transcription rates (**A**) and expression levels (**B**) of *TaCYP86A2*, *TaCYP86A4*, and *TaECR* in the wheat leaves with silenced gene *TaTCP15* were measured by nuclear run-on and qRT-PCR assays, respectively. (**C**) Total cutin and cuticular wax amounts in the *TaTCP15*-silenced wheat leaves. (**D**) Amounts of major cutin monomers in the *TaTCP15*-silenced wheat leaves. (**E**) Amounts of major wax components in the *TaTCP15*-silenced wheat leaves. (**F**) Water loss rates and (**G**) chlorophyll extraction levels analyzed in *TaTCP15*-silenced wheat leaves. BSMV-γ was employed as the negative control, and three technical replicates per treatment were statistically analyzed by Student’s *t*-test (** represents *p* < 0.01).

As shown in Figure 6A,B, silencing of the *TaTCP15* gene resulted in a significant reduction in the transcription rates and expression levels of *TaCYP86A2*, *TaCYP86A4*, and *TaECR* genes. GC-MS assay demonstrated that silencing of the *TaTCP15* gene resulted in a significant reduction in the loads of total cutin monomers and cuticular wax in the wheat leaves (Figure 6C). Subsequent cutin monomer composition and wax constituent analyses revealed that cutin monomers and major wax constituents showed a significant reduction in the wheat leaves with silenced gene *TaTCP15* (Figure 6D,E). Water loss rate and chlorophyll leaching analysis validated that cuticle permeability was enhanced on the wheat leaves with silenced gene *TaTCP15* (Figure 6F,G). These results suggest that wheat transcriptional activator TaTCP15 stimulates the biosynthesis of cutin and cuticular wax, essential for the surface barrier property of cuticle.

## 4. Discussion

### 4.1. CYP86A Family Cytochrome P450 Enzyme TaCYP86A2 and TaCYP86A4 Proteins Show Partially Redundant Contributions to Wheat Cutin Biosynthesis

CYP86A family cytochrome P450 enzymes catalyze the oxidation reactions in the production of cutin monomers [19]. The Arabidopsis *cyp86a2* mutant displayed a 70% reduction in total cutin loads, contributed by decreased accumulation of cutin monomer components 9-hydroxy pentadecanoic acid, 10(9)-hydroxy heptadecanoic acid, 16-hydroxy hexadecanoic acid, 10,16-dihydroxy hexadecanoic acid, hexadecane-1,16-dioic acid, 7-hydroxy hexadecane-1,16-dioic acid, and octadecane-1,18-dioic acid. Similarly, the Arabidopsis *cyp86a4* mutant exhibited a 34% reduction in total cutin loads [19]. TaCYP86A2 was previously characterized as a core component of wheat cutin biosynthetic machinery, and silencing of the *TaCYP86A2* gene resulted in reduced loads of cutin monomers [49]. However, the potential contribution of the *CYP86A4* gene to wheat cutin biosynthesis remains unknown. Herein, we identified the wheat *TaCYP86A4* gene as a homolog of the Arabidopsis *AtCYP86A4* gene. The silencing of *TaCYP86A2* and *TaCYP86A4* genes resulted in a reduction in total cutin loads to 45.2% and 57.4%, respectively, and simultaneously silencing all endogenous *TaCYP86A2* and *TaCYP86A4* genes resulted in a further reduction in total cutin loads to 22.6%. Notably, silencing of *TaCYP86A2* and *TaCYP86A4* genes resulted in decreased loads of all cutin monomer components, including 16-hydroxy-hexadecanoic acid, 18-hydroxy-octadec-9-enoic acid, 9,10-epoxy 18-hydroxy-octadecanoic acid, 9(10), 16-dihydroxy-hexadecanoic acid, and 9,10,18-trihydroxy-octadecanoic acid, suggesting that TaCYP86A2 and TaCYP86A4 proteins show partially redundant contributions to wheat cutin biosynthesis.

### 4.2. Wheat Transcription Factor TaSHN1 and Its Interactor Mediator Subunit TaCDK8 Directly Regulate Transcription of Cutin Biosynthesis Genes TaCYP86A2 and TaCYP86A4

Wheat transcription factor TaSHN1 was previously identified as a key regulator of cutin and cuticular wax biosynthesis [48]. The mediator kinase subunit TaCDK8 was demonstrated to interact with and phosphorylate TaSHN1 to potentiate biosynthesis of cutin and cuticular wax [48]. However, the wheat cuticle biosynthesis genes directly targeted by the TaCDK8-TaSHN1 module remain unknown. In this study, we employed ChIP-qPCR and the promoter activation assay to demonstrate that transcriptional activator TaSHN1 directly binds to promoter regions of cutin biosynthesis genes *TaCYP86A2* and *TaCYP86A4* and wax biosynthesis gene *TaECR* by recognizing the *GCC*-box cis-element. ChIP-qPCR and wheat protoplast transactivation assays further revealed that transcription factor TaSHN1 could recruit mediator kinase subunit TaCDK8 to activate transcription of *TaCYP86A2*, *TaCYP86A4*, and *TaECR* genes. Notably, coexpression of TaSHN1 with wild-type TaCDK8, but not the TaCDK8(D176A) mutant with defective kinase activity, resulted in a further increase in the LUC activity obtained from the reporter gene driven by *TaCYP86A2*, *TaCYP86A4*, and *TaECR* promoters in the wheat protoplast transactivation assay, suggesting that TaCDK8-mediated TaSHN1 phosphorylation might be essential for the transcriptional activation of *TaCYP86A2*, *TaCYP86A4*, and *TaECR* genes mediated by the TaCDK8-TaSHN1 module. These studies collectively suggested that wheat transcription factor TaSHN1 and its interactor mediator subunit TaCDK8 positively regulate cutin and cuticular wax biosynthesis by activating cutin biosynthesis genes *TaCYP86A2* and *TaCYP86A4* and wax biosynthesis gene *TaECR*. Previous studies revealed that transcription of *TaCYP86A2* and *TaECR* genes could be regulated by Topoisomerase VI complex and SAGA histone acetyltransferase complex, respectively [49,50]. Therefore, it will be intriguing to examine the potential interplays among the TaCDK8-TaSHN1 module, Topoisomerase VI complex, and SAGA histone acetyltransferase complex in the transcriptional regulation of cuticle biosynthesis in future research.

### 4.3. Wheat TCP-Type Transcription Factor TaTCP15 Transactivates the TaSHN1 Gene and Stimulates Cutin and Wax Biosynthesis

The Arabidopsis *AtSHN1* gene was previously demonstrated to be directly activated by the TCP transcription factor AtTCP15 [56]. However, the mechanism underlying the transcriptional regulation of the *SHN1* gene in other plant species, especially in the agronomically important crop bread wheat, remains unknown. By employing the ChIP and wheat protoplast transactivation assay, we demonstrated that transcription factor TaTCP15 could bind to *TaSHN1* promoters by recognizing the *TCP box* cis-element and directly activate *TaSHN1* transcription. Reduced *TaSHN1* expression and attenuated cutin and wax accumulation were observed in the *TaTCP15*-silenced wheat plant, confirming that the transcription factor TaTCP15 positively regulates cutin and wax biosynthesis, probably via transactivation of *TaSHN1* genes. In the dicot model plant *A. thaliana*, expression of the *AtSHN1* gene is directly activated by the TCP-type transcription factor AtTCP15. *AtTCP15* gene loss-of-function plants *tcp15-3* displayed increased cuticle permeability [50]. These studies suggested that transcriptional activation of *SHN1* genes, as well as cutin biosynthesis by TCP15, might be conserved among the dicot model plant *A. thaliana* and monocot cereal crop bread wheat.

Although some of the genes we identified in wheat are homologs of *Arabidopsis* genes that have been previously characterized, our paper reported for the first time that the class 1 type transcription factor TaTCP15 stimulates wheat cutin and wax biosynthesis by activating transcription of the *TaSHN1* gene. We firstly identified partially redundant TaCYP86A2 and TaCYP86A4 proteins as key components of wheat cutin biosynthetic machinery and then elucidated that the transcription factor TaSHN1 recruits the mediator subunit TaCDK8 to activate transcription of *TaCYP86A2*, *TaCYP86A4*, and wax biosynthesis gene *TaECR*. Finally, we demonstrated that TaTCP15 stimulates wheat cutin and wax biosynthesis by activating transcription of the *TaSHN1* gene. Our study uncovers for the first time the regulatory role of the TaTCP15-TaSHN1-*TaCYP86A2*/*TaCYP86A4*/*TaECR* circuit in wheat cutin and wax biosynthesis.

Cutin and wax are major lipophilic components of the cuticle, which contributes to plant adaptation to environmental stresses. Genetic manipulation of *TaCYP86A2*, *TaCYP86A4*, and *TaECR* genes, as well as their regulator genes like *TaTCP15* and *TaSHN1*, by CRISPR (clustered regularly interspaced short palindromic repeats)-Cas9 (CRISPR-associated nuclease 9), or targeting induced local lesions in genomes (TILLING) might provide a new avenue for improving wheat resistance against environmental stresses. In addition, the regulation of cutin and cuticular wax biosynthesis is complicated, often organ-dependent, and influenced by environmental conditions. Therefore, exploring the regulatory effect of the TaTCP15-TaSHN1-*TaCYP86A2*/*TaCYP86A4*/*TaECR* circuit on cuticle biosynthesis in other tissues, like leaf sheaths, stems, or spikelets, as well as their response to developmental and environmental cues, could provide novel insights into the plant cuticle biosynthetic mechanisms in future research.

## 5. Conclusions

In this study, we revealed that the wheat class I TCP transcription factor TaTCP15 directly activates transcription of *TaSHN1*, a transcriptional activator gene of wheat cutin and wax biosynthesis. We found that wheat transcription factor TaSHN1 targets *TaCYP86A2*, *TaCYP86A4*, and wax biosynthesis gene *TaECR* and recruits the mediator subunit TaCDK8 to activate these genes’ transcription. Furthermore, we demonstrated that the expression of *TaSHN1*, *TaCYP86A2*, *TaCYP86A4*, and *TaECR* genes, as well as cutin and wax accumulation, was attenuated by silencing of the *TaTCP15* gene. Collectively, these findings suggest that wheat class I TCP transcription factor TaTCP15 positively regulates cutin and cuticular wax biosynthesis, probably by directly targeting the *TaSHN1* gene and upregulating *TaCYP86A2*, *TaCYP86A4*, and *TaECR* expression. This study revealed a novel regulatory mechanism underlying wheat cuticle biosynthesis and provided valuable information for developing wheat plants with improved cuticle-associated traits.

## Data Availability

The original contributions presented in this study are included in the article/Supplementary Material. Further inquiries can be directed to the corresponding author(s).

## Supplementary Materials

The following supporting information can be downloaded at https://www.mdpi.com/article/10.3390/biom16020192/s1: Supplementary Figure S1. Transactivation activity analysis of TaTCP15 in wheat protoplast cells. Supplementary Table S1. Primers used in this study.
