# Supplementary material for: Wheat Class I TCP Transcription Factor TaTCP15 Positively Regulates Cutin and Cuticular Wax Biosynthesis
Source: Biomolecules. 2026 Jan 27;16(2):192. doi: 10.3390/biom16020192 (PMC12938820; doi:10.3390/biom16020192)
Supplement: Supplementary file 1 [file biomolecules-16-00192-s001.zip › biomolecules-4084138-supplementary.pdf]

Supplementary Data

Wheat Class I TCP transcription factor TaTCP15 positively regulates cutin and cuticular wax biosynthesis

Linzhu Fang, Xiaoyu Wang, Haoyu Li, Jiao Liu, Pengfei Zhi, and Cheng Chang*

College of Life Sciences, Qingdao University, Qingdao 266071, China

* Corresponding author: Cheng Chang (cc@qdu.edu.cn)

Table of Contents:

Supplementary Figure S1 Transactivation activity analysis of TaTCP15 in wheat protoplast cells

Supplementary Table S1 Primers used in this study.

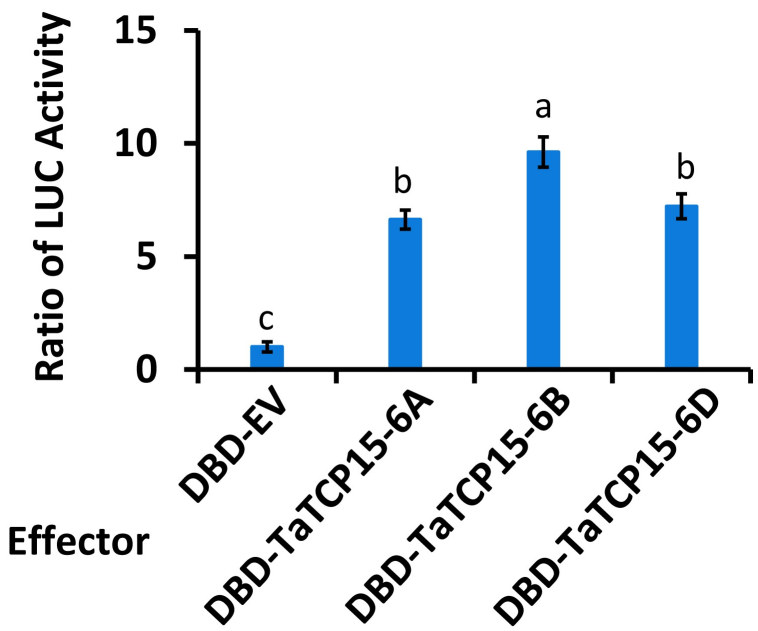

Supplementary Figure S1 Transactivation activity analysis of TaTCP15. The LUC reporter used in this assay contains 5x Gal4 UAS. Three technical replicates per treatment were statistically analyzed by One-way ANOVA analysis (different letters represent $P<0.05$), and these assays were repeated in three independent biological replicates with similar results.

Supplemental Table S1 Primers used in this study.

**Primer Name**	**Sequence**	**Annotation**
qRT-PCR-*TaCYP86A2*-F	5' GACGCGGCCAAGGGCAAG3'	qRT-PCR primer for *TaCYP86A2*, F primer
qRT-PCR-*TaCYP86A2*-R	5' TGCGTCGCCGCCGTGTCGC3'	qRT-PCR primer for *TaCYP86A2*, R primer
qRT-PCR-*TaCYP86A4*-F	5' GTCCGACGCGGCGGCCTCC3'	qRT-PCR primer for *TaCYP86A4*, F primer
qRT-PCR-*TaCYP86A4*-R	5' GTGGACCCGTCGCTCTTGC3'	qRT-PCR primer for *TaCYP86A4*, R primer
qRT-PCR-*TaECR*-F	5' TGAAGGTCTCCGTCGTGTCC3′	qRT-PCR primer for *TaECR*, F primer
qRT-PCR-*TaECR*-R	5' CAGAAGAAGAGCGTGCTGTAG3′	qRT-PCR primer for *TaECR*, R primer
qRT-PCR-*TaSHN1*-F	5' GGTGCAACCCAAGAAGAAG3'	qRT-PCR primer for *TaSHN1*, F primer
qRT-PCR-*TaSHN1*-R	5' ATCGCCGGTGCTGCTCCTC3'	qRT-PCR primer for *TaSHN1*, R primer
qRT-PCR-*TaTCP15*-F	5' GAGCCCCGGCGTAAGGTTC3'	qRT-PCR primer for *TaTCP15*, F primer
qRT-PCR-*TaTCP15*-R	5' TCACCACTTCCTCCTCCAC3'	qRT-PCR primer for *TaTCP15*, R primer
BSMV-*TaCYP86A2as*-F	5'AAGGAAGTTTAACATGCACCCGTGCTCTGAG3'	For construct of BSMV-*TaCYP86A2as*, F primer
BSMV-*TaCYP86A2as*-R	5'AACCACCACCACCGTTGCTAGCTGTTGAAGGGAC3'	For construct of BSMV-*TaCYP86A2as*, R primer
BSMV-*TaCYP86A4as*-F	5'AAGGAAGTTTAAACAGTACAGTACAGGAGAG3'	For construct of BSMV-*TaCYP86A4as*, F primer
BSMV-*TaCYP86A4as*-R	5'AACCACCACCACCGTCATGCATGCTCCACGTACG3'	For construct of BSMV-*TaCYP86A4as*, R primer
BSMV-*TaSHN1as*-F	5'AAGGAAGTTTAAAGACGACGAAGCTACCTTC3'	For construct of BSMV-*TaSHN1as*, F primer
BSMV-*TaSHN1as*-R	5'AACCACCACCACCGTGTCTGGCAGAAGCGTGCAG3'	For construct of BSMV-*TaSHN1as*, R primer
BSMV-*TaCDK8as*-F	5'AAGGAAGTTTAAAGAAGCGCCTCTTCTGCTG3'	For construct of BSMV-*TaCDK8as*, F primer
BSMV-*TaCDK8as*-R	5'AACCACCACCACCGTCAAATCCCAAGGCAAATGC3'	For construct of BSMV-*TaCDK8as*, R primer
BSMV-*TaTCP15as*-F	5'AAGGAAGTTTAACGGGATGGAGAGCGAGGAG3'	For construct of BSMV-*TaTCP15as*, F primer
BSMV-*TaTCP15as*-R	5'AACCACCACCACCGTGCAGGTGCGGAAGGCGGTG3'	For construct of BSMV-*TaTCP15as*, R primer
ChIP-qPCR-*TaCYP86A2-6A*-1-F	5'CACTCAACGTACCACCTGC3'	ChIP-qPCR primer for *TaCYP86A2-6A*-1, F primer
ChIP-qPCR-*TaCYP86A2-6A*-1-R	5'GGGTATCTATTTTGAGGTG3'	ChIP-qPCR primer for *TaCYP86A2-6A*-1, R primer
ChIP-qPCR-*TaCYP86A2-6A*	5'GTGACCGGGCTCTGGAGTC3'	ChIP-qPCR primer for *TaCYP86A2-6A*-2, F

-2-F		primer
ChIP-qPCR-*TaCYP86A2-6A*-2-R	5'GCGATTGGCCTCGGCTGAC3'	ChIP-qPCR primer for *TaCYP86A2-6A*-2, R primer
ChIP-qPCR-*TaCYP86A2-6B*-1-F	5'CTTCTCTGAACCGCTCAACG 3'	ChIP-qPCR primer for *TaCYP86A2-6B*-1, F primer
ChIP-qPCR-*TaCYP86A2-6B*-1-R	5'CAGGTTCGACAAGAAAATC3 '	ChIP-qPCR primer for *TaCYP86A2-6B*-1, R primer
ChIP-qPCR-*TaCYP86A2-6B*-2-F	5'GACACGATCGAAGCGGAAA C3'	ChIP-qPCR primer for *TaCYP86A2-6B*-2, F primer
ChIP-qPCR-*TaCYP86A2-6B*-2-R	5'CCAAGGCCCAACCAAGACG 3'	ChIP-qPCR primer for *TaCYP86A2-6B*-2, R primer
ChIP-qPCR-*TaCYP86A2-6D*-1-F	5'CACTATTACAGTGTTCAAAA C3'	ChIP-qPCR primer for *TaCYP86A2-6D*-1, F primer
ChIP-qPCR-*TaCYP86A2-6D*-1-R	5'CAAAAAGGCGGGTTGCAAT C3'	ChIP-qPCR primer for *TaCYP86A2-6D*-1, R primer
ChIP-qPCR-*TaCYP86A2-6D*-2-F	5'CTTTGCGTGTGTGTGTAGTG3'	ChIP-qPCR primer for *TaCYP86A2-6D*-2, F primer
ChIP-qPCR-*TaCYP86A2-6D*-2-R	5'TGGTTTCTCCTTTGCTTGG3'	ChIP-qPCR primer for *TaCYP86A2-6D*-2, R primer
ChIP-qPCR-*TaCYP86A4-2A*-1-F	5'GATGGAGTCAGATGTTGTAG 3'	ChIP-qPCR primer for *TaCYP86A4-2A*-1, F primer
ChIP-qPCR-*TaCYP86A4-2A*-1-R	5'CAAAGCTAATGCAGCAGGTA 3'	ChIP-qPCR primer for *TaCYP86A4-2A*-1, R primer
ChIP-qPCR-*TaCYP86A4-2A*-2-F	5'ACGCAGGGCGGATGGGGTA C3'	ChIP-qPCR primer for *TaCYP86A4-2A*-2, F primer
ChIP-qPCR-*TaCYP86A4-2A*-2-R	5'CAAGGCGATGGATGGCAAT G3'	ChIP-qPCR primer for *TaCYP86A4-2A*-2, R primer
ChIP-qPCR-*TaCYP86A4-2B*-1-F	5'TTGTAGATAAATGTTGGGTG3 '	ChIP-qPCR primer for *TaCYP86A4-2B*-1, F primer
ChIP-qPCR-*TaCYP86A4-2B*-1-R	5'CAAAACGGCATTACTATCAG 3'	ChIP-qPCR primer for *TaCYP86A4-2B*-1, R primer
ChIP-qPCR-*TaCYP86A4-2B*-2-F	5' CTCCATATATCTCGCGCACA3'	ChIP-qPCR primer for *TaCYP86A4-2B*-2, F primer
ChIP-qPCR-*TaCYP86A4-2B*-2-R	5' AGGACGAGGCTATATAGAAG3'	ChIP-qPCR primer for *TaCYP86A4-2B*-2, R primer
ChIP-qPCR-*TaCYP86A4-2D*-1-F	5' CAGTCCGATGGAGTCAGATC3'	ChIP-qPCR primer for *TaCYP86A4-2D*-1, F primer
ChIP-qPCR-*TaCYP86A4-2D*-1-R	5' TAGATGCATGGGTGAAACTG3'	ChIP-qPCR primer for *TaCYP86A4-2D*-1, R primer
ChIP-qPCR-*TaCYP86A4-2D*-2-F	5' CACTGCCTTGGCCTACTCC3'	ChIP-qPCR primer for *TaCYP86A4-2D*-2, F primer
ChIP-qPCR-*TaCYP86A4-2D*-2-R	5' GGACGGGGCTATATAGAAGC3'	ChIP-qPCR primer for *TaCYP86A4-2D*-2, R primer

ChIP-qPCR-*TaECR-3A*-1-F	5'GCAAGTGCATCATGCGATG3'	ChIP-qPCR primer for *TaECR-3A*-1, F primer
ChIP-qPCR-*TaECR-3A*-1-R	5'GAGGGTCTCAGTTGGTTGG3'	ChIP-qPCR primer for *TaECR-3A*-1, R primer
ChIP-qPCR-*TaECR-3A*-2-F	5'GGGATCTCACTCAAATACG3'	ChIP-qPCR primer for *TaECR-3A*-2, F primer
ChIP-qPCR-*TaECR-3A*-2-R	5'CTCCCTCCGCTGGTGGCTTC3'	ChIP-qPCR primer for *TaECR-3A*-2, R primer
ChIP-qPCR-*TaECR-3B*-1-F	5' GTGTGCAAGTGCATGCGATG3'	ChIP-qPCR primer for *TaECR-3B*-1, F primer
ChIP-qPCR-*TaECR-3B*-1-R	5' GGACTGGGTTCAGCACCACG3'	ChIP-qPCR primer for *TaECR-3B*-1, R primer
ChIP-qPCR-*TaECR-3B*-2-F	5' CACAATGAAGTAGACAAAAC 3'	ChIP-qPCR primer for *TaECR-3B*-2, F primer
ChIP-qPCR-*TaECR-3B*-2-R	5' CTACCACGTACACAACTCT3'	ChIP-qPCR primer for *TaECR-3B*-2, R primer
ChIP-qPCR-*TaECR-3D*-1-F	5' GAAACCGGAACGGTGCCAGT 3'	ChIP-qPCR primer for *TaECR-3D*-1, F primer
ChIP-qPCR-*TaECR-3D*-1-R	5' GGCGACGGACGTCAGCGAG3'	ChIP-qPCR primer for *TaECR-3D*-1, R primer
ChIP-qPCR-*TaECR-3D*-2-F	5' AACCTACAACTCCAAACATAC 3'	ChIP-qPCR primer for *TaECR-3D*-2, F primer
ChIP-qPCR-*TaECR-3D*-2-R	5' CTCACCACCGACAACCGAGG3'	ChIP-qPCR primer for *TaECR-3D*-2, R primer
ChIP-qPCR-*TaSHN1-6A*-1-F	5' ACACAAGTCTTACCATATTG3'	ChIP-qPCR primer for *TaSHN1-6A* -1, F primer
ChIP-qPCR-*TaSHN1-6A*-1-R	5' GAGCTCGCCATCTTGGTTC3'	ChIP-qPCR primer for *TaSHN1-6A* -1, R primer
ChIP-qPCR-*TaSHN1-6A*-2-F	5' GTACCACCACCAGTCTAGC3'	ChIP-qPCR primer for *TaSHN1-6A* -2, F primer
ChIP-qPCR-*TaSHN1-6A*-2-R	5' GTGTTTATTACCACTGTAC3'	ChIP-qPCR primer for *TaSHN1-6A* -2, R primer
ChIP-qPCR-*TaSHN1-6B*-1-F	5' CTTACCATATTGACAATCT3'	ChIP-qPCR primer for *TaSHN1-6B* -1, F primer
ChIP-qPCR-*TaSHN1-6B*-1-R	5' CAGAAAGAGAGCTCACCATC3'	ChIP-qPCR primer for *TaSHN1-6B* -1, R primer
ChIP-qPCR-*TaSHN1-6B*-2-F	5' GGCAGAAGACAAGAGCGAG3'	ChIP-qPCR primer for *TaSHN1-6B* -2, F primer
ChIP-qPCR-*TaSHN1-6B*-2-R	5' CAATTAAATGTGGCTGGCC3'	ChIP-qPCR primer for *TaSHN1-6B* -2, R primer
ChIP-qPCR-*TaSHN1-6D*-1-F	5' CAGCAAGTCTTACTCAAAAC3'	ChIP-qPCR primer for *TaSHN1-6D* -1, F primer
ChIP-qPCR-*TaSHN1-6D*-1-	5' CTTTGTGTGCGATGGCCTC3'	ChIP-qPCR primer for *TaSHN1-6D* -1, R primer

R		
ChIP-qPCR-*TaSHN1-6D*-2-F	5' GCAAATGATTTAATGAAGC3'	ChIP-qPCR primer for *TaSHN1-6D* -2, F primer
ChIP-qPCR-*TaSHN1-6D*-2-R	5' CATTGGAAGGCAGTGTATAC3'	ChIP-qPCR primer for *TaSHN1-6D* -2, R primer
ChIP-qPCR-*TaTCP15-6A*-1-F	5' GTGCACAGTGGCAATAGTG3'	ChIP-qPCR primer for *TaTCP15-6A* -1, F primer
ChIP-qPCR-*TaTCP15-6A*-1-R	5' CCTAGCCAGCCATGGCGTG3'	ChIP-qPCR primer for *TaTCP15-6A* -1, R primer
ChIP-qPCR-*TaTCP15-6A*-2-F	5' CTACCGTCGTCTCCATCAC3'	ChIP-qPCR primer for *TaTCP15-6A* -2, F primer
ChIP-qPCR-*TaTCP15-6A*-2-R	5' GTCAATTAGGCCATGTTAT3'	ChIP-qPCR primer for *TaTCP15-6A* -2, R primer
ChIP-qPCR-*TaTCP15-6B*-1-F	5' GTAGCAATAGTGCTATGGTG3'	ChIP-qPCR primer for *TaTCP15-6B* -1, F primer
ChIP-qPCR-*TaTCP15-6B*-1-R	5' CTCTCTGTGCTGGCTAGCT3'	ChIP-qPCR primer for *TaTCP15-6B* -1, R primer
ChIP-qPCR-*TaTCP15-6B*-2-F	5' CCATCACACCTAACCAATC3'	ChIP-qPCR primer for *TaTCP15-6B* -2, F primer
ChIP-qPCR-*TaTCP15-6B*-2-R	5' ATTAGGCCATGTTATATAC3'	ChIP-qPCR primer for *TaTCP15-6B* -2, R primer
ChIP-qPCR-*TaTCP15-6D*-1-F	5' GTTAATCCCTAGCGTGCAC3'	ChIP-qPCR primer for *TaTCP15-6D* -1, F primer
ChIP-qPCR-*TaTCP15-6D*-1-R	5' GCTCACCTAGCCCGCCATG'	ChIP-qPCR primer for *TaTCP15-6D* -1, R primer
ChIP-qPCR-*TaTCP15-6D*-2-F	5' CATCACACCTAACCAATCT3'	ChIP-qPCR primer for *TaTCP15-6D* -2, F primer
ChIP-qPCR-*TaTCP15-6D*-2-R	5' TAGGCCATGTTATATACAC3'	ChIP-qPCR primer for *TaTCP15-6D* -2, R primer
pENTRY-*proTaCYP86A2-6A*-F	5'GGGGACAAGTTTGTACAAAAAAGCAGGCTTCGCCTAATCCACAACTTATC3 '	For the construction of pENTRY-*proTaCYP86A2-6A*, F primer
pENTRY-*proTaCYP86A2-6A*-R	5'GGGGACCACTTTGTACAAGAAAGCTGGGTCTGCCGGCTGACCTCCCTGA3'	For the construction of pENTRY-*proTaCYP86A2-6A*, R primer
pENTRY-*proTaCYP86A2-6B*-F	5'GGGGACAAGTTTGTACAAAAAAGCAGGCTTCCTATTTTTTGGCCAATTG3 '	For the construction of pENTRY-*proTaCYP86A2-6B*, F primer
pENTRY-*proTaCYP86A2-6B*-R	5'GGGGACCACTTTGTACAAGAAAGCTGGGTCTGCTGGCCTCCCTGGAGCA3'	For the construction of pENTRY-*proTaCYP86A2-6B*, R primer
pENTRY-*proTaCYP86A2-6D*-F	5'GGGGACAAGTTTGTACAAAAAAGCAGGCTTCCCAATCATATCTTTTACAC3 '	For the construction of pENTRY-*proTaCYP86A2-6B*, F primer

pENTRY-*proTaCYP86A2-6D*-R	5'GGGGACCACTTTGTACAAGAAAGCTGGGTCTGCCGGCCTCCCTGAAGCAAG3'	For the construction of pENTRY-*proTaCYP86A2-6B*, R primer
pENTRY-*proTaCYP86A4-2A*-F	5'GGGGACAAGTTTGTACAAAAAAGCAGGCTTCTATTCTCAGAACAAATCCCTC3 '	For the construction of pENTRY-*proTaCYP86A4-2A*, F primer
pENTRY-*proTaCYP86A4-2A*-R	5'GGGGACCACTTTGTACAAGAAAGCTGGGTCCGCTAAGCTGGCCAAGCTCGA3'	For the construction of pENTRY-*proTaCYP86A4-2A*, R primer
pENTRY-*proTaCYP86A4-2B*-F	5'GGGGACAAGTTTGTACAAAAAAGCAGGCTTCGGTGGACATGTTCTTGAGG3 '	For the construction of pENTRY-*proTaCYP86A4-2B*, F primer
pENTRY-*proTaCYP86A4-2B*-R	5'GGGGACCACTTTGTACAAGAAAGCTGGGTCCGCCAAGCTAGCCAAGCTC3'	For the construction of pENTRY-*proTaCYP86A4-2B*, R primer
pENTRY-*proTaCYP86A4-2D*-F	5'GGGGACAAGTTTGTACAAAAAAGCAGGCTTCGAATTACTTGGCACAATATTC3 '	For the construction of pENTRY-*proTaCYP86A4-2B*, F primer
pENTRY-*proTaCYP86A4-2D*-R	5'GGGGACCACTTTGTACAAGAAAGCTGGGTCCGCCAAGCTAGCCAAGCTCG3'	For the construction of pENTRY-*proTaCYP86A4-2B*, R primer
pENTRY-*proTaECR-3A*-F	5'GGGGACAAGTTTGTACAAAAAAGCAGGCTTCTTGGTGTTCGTTTATCCATT3 '	For the construction of pENTRY-*proTaECR-3A*, F primer
pENTRY-*proTaECR-3A*-R	5'GGGGACCACTTTGTACAAGAAAGCTGGGTCGCTCCCTCCGCTGGTGGCTTC3'	For the construction of pENTRY-*proTaECR-3A*, R primer
pENTRY-*proTaECR-3B*-F	5'GGGGACAAGTTTGTACAAAAAAGCAGGCTTCCCTTAGGGTGATGCGCCTAC3 '	For the construction of pENTRY-*proTaECR-3B*, F primer
pENTRY-*proTaECR-3B*-R	5'GGGGACCACTTTGTACAAGAAAGCTGGGTCGCTCCCTCCGCCGGTGGCTTC3'	For the construction of pENTRY-*proTaECR-3B*, R primer
pENTRY-*proTaECR-3D*-F	5'GGGGACAAGTTTGTACAAAAAAGCAGGCTTCTGGACGCGCAGCCCCTCCGGT3 '	For the construction of pENTRY-*proTaECR-3B*, F primer
pENTRY-*proTaECR-3D*-R	5'GGGGACCACTTTGTACAAGAAAGCTGGGTCGCTCCCTCCGCTGGTGGATTC3'	For the construction of pENTRY-*proTaECR-3B*, R primer
pENTRY-*proTaSHN1-6A*-F	5'GGGGACAAGTTTGTACAAAAAAGCAGGCTTCGCACGTAGGCATATCCATATG3 '	For the construction of pENTRY-*proTaSHN1-6A*, F primer
pENTRY-*proTaSHN1-6A*-R	5'GGGGACCACTTTGTACAAGAAAGCTGGGTCTTGTTCTGCCT	For the construction of pENTRY-*proTaSHN1-6A*, R primer

	GTCTTTCAC3'	
pENTRY-*proTaSHN1-6B*-F	5'GGGGACAAGTTTGTACAAAAAAGCAGGCTTCCATATCCATATGCAGTATGC3 '	For the construction of pENTRY-*proTaSHN1-6B*, F primer
pENTRY-*proTaSHN1-6B*-R	5'GGGGACCACTTTGTACAAGAAAGCTGGGTCTTTGTTCTGTCTGTCTGTCC3'	For the construction of pENTRY-*proTaSHN1-6B*, R primer
pENTRY-*proTaSHN1-6D*-F	5'GGGGACAAGTTTGTACAAAAAAGCAGGCTTCCACACATATTAACACTCGAA3 '	For the construction of pENTRY-*proTaSHN1-6D*, F primer
pENTRY-*proTaSHN1-6D*-R	5'GGGGACCACTTTGTACAAGAAAGCTGGGTCTTGTTCTGCCTGCCTGTCTTTC3'	For the construction of pENTRY-*proTaSHN1-6D*, R primer
pENTRY-*proTaTCP15-6A*-F	5'GGGGACAAGTTTGTACAAAAAAGCAGGCTTCTGGTGAATTTCTATCTACCAA3 '	For the construction of pENTRY-*proTaTCP15-6A*, F primer
pENTRY-*proTaTCP15-6A*-R	5'GGGGACCACTTTGTACAAGAAAGCTGGGTCGGTCAATTAGGCCATGTTAT3'	For the construction of pENTRY-*proTaTCP15-6A*, R primer
pENTRY-*proTaTCP15-6B*-F	5'GGGGACAAGTTTGTACAAAAAAGCAGGCTTCAGTTAATTGTTCTTTTGAGA3 '	For the construction of pENTRY-*proTaTCP15-6B*, F primer
pENTRY-*proTaTCP15-6B*-R	5'GGGGACCACTTTGTACAAGAAAGCTGGGTCGGTCAATTAGGCCATGTTAT3'	For the construction of pENTRY-*proTaTCP15-6B*, R primer
pENTRY-*proTaTCP15-6D*-F	5'GGGGACAAGTTTGTACAAAAAAGCAGGCTTCCAAAAAAAACATGCATAGTTG3 '	For the construction of pENTRY-*proTaTCP15-6D*, F primer
pENTRY-*proTaTCP15-6D*-R	5'GGGGACCACTTTGTACAAGAAAGCTGGGTCGGTCAATTAGGCCATGTTAT3'	For the construction of pENTRY-*proTaTCP15-6D*, R primer
pENTRY-*TaSHN1*-F	5'GGGGACAAGTTTGTACAAAAAAGCAGGCTTCATGGTGCAACCCAAGAAGA3 '	For the construction of pENTRY-*TaSHN1*, F primer
pENTRY-*TaSHN1*-R	5'GGGGACCACTTTGTACAAGAAAGCTGGGTCGACGACGAAGCTACCTTCT3'	For the construction of pENTRY-*TaSHN1*, R primer
pENTRY-*TaCDK8*-F	5'GGGGACAAGTTTGTACAAAAAAGCAGGCTTCATGGGCGACGGCCGCGGCG3 '	For the construction of pENTRY-*TaCDK8*, F primer
pENTRY-*TaCDK8*-R	5'GGGGACCACTTTGTACAAGAAAGCTGGGTCGAAGCGCCTCTTCTGCTGA3'	For the construction of pENTRY-*TaCDK8*, R primer
pENTRY-*TaTCP15*-F	5'GGGGACAAGTTTGTACAAA	For the construction of pENTRY-*TaTCP15*, F

	AAAGCAGGCTTCATGGACATCGCCGGAGACG3 '	primer
pENTRY-*TaTCP15*-R	5'GGGGACCACTTTGTACAAGAAAGCTGGGTCCGACTCGCTGGTGCTCATG3'	For the construction of pENTRY-*TaTCP15*, R primer
pENTRY-RNAi-*TaSHN1as*-F	5'GGGGACAAGTTTGTACAAAAAAGCAGGCTTCAGACGACGAAGCTACCTTC3'	For the construction of pENTRY-RNAi-*TaSHN1as*, F primer
pENTRY-RNAi-*TaSHN1as*-R	5'GGGGACCACTTTGTACAAGAAAGCTGGGTCGTCTGGCAGAAGCGTGCAG3'	For the construction of pENTRY-RNAi-*TaSHN1as*, R primer
pENTRY-RNAi-*TaTCP15as*-F	5'GGGGACAAGTTTGTACAAAAAAGCAGGCTTCCGGGATGGAGAGCGAGGAG3'	For the construction of pENTRY-RNAi-*TaTCP15as*, F primer
pENTRY-RNAi-*TaTCP15as*-R	5'GGGGACCACTTTGTACAAGAAAGCTGGGTCGCAGGTGCGGAAGGCGGTG3'	For the construction of pENTRY-RNAi-*TaTCP15as*, R primer
